# A novel, robust method for quantification of multiple kynurenine pathway metabolites in the cerebrospinal fluid

**DOI:** 10.4155/bio-2019-0303

**Published:** 2020-03-25

**Authors:** Lilly Schwieler, Ada Trepci, Stanislaw Krzyzanowski, Sigurd Hermansson, Mathias Granqvist, Fredrik Piehl, Tomas Venckunas, Marius Brazaitis, Sigitas Kamandulis, Daniel Lindqvist, Arthur Daniel Jones, Sophie Erhardt, Lena Brundin

**Affiliations:** 1 Department of Physiology & Pharmacology, Karolinska Institutet, Stockholm 17165, Sweden; 2 Institute of Sport Science & Innovations, Lithuanian Sports University, Kaunas 44221, Lithuania; 3 Center for Neurodegenerative Science, Van Andel Research Institute, Grand Rapids, MI 49503, USA; 4 Waters Sverige AB, Stockholm 17165, Sweden; 5 Unit of Neuroimmunology, Department of Clinical Neuroscience, Center for Molecular Medicine, Karolinska Institutet, Karolinska University Hospital, Stockholm 171 77, Sweden; 6 Division of Neurology, Karolinska University Hospital, Stockholm 171 76, Sweden; 7 Department of Clinical Sciences Lund, Faculty of Medicine, Lund University, Psychiatry, Lund 221 84, Sweden; 8 Department of Biochemistry & Molecular Biology & Department of Chemistry & Director, RTSF/MSU Mass Spectrometry & Metabolomics Core, Michigan State University, East Lansing, MI 48824, USA

**Keywords:** LC–MS/MS, nicotinic acid, picolinic acid, quinolinic acid, suicide, tryptophan-kynurenine pathway

## Abstract

**Aim:**

Kynurenine metabolites are potential modulators of psychiatric disease. We aimed to develop a highly sensitive biochemical analysis of cerebrospinal fluid (CSF) tryptophan (TRP) metabolites, to investigate the stability of metabolites and to confirm our previous findings of aberrant CSF quinolinic acid (QUIN) and picolinic acid (PIC) in suicide attempters using this method.

**Methodology & results:**

Ten CSF TRP metabolites were analyzed with ultraperformance LC–MS/MS. The method showed small intra- and interassay variation. Metabolites were stable following freeze–thaw cycles. A decreased CSF PIC/QUIN ratio was found in suicide attempters.

**Conclusion:**

The feasibility of reliably determining CSF TRP metabolites were demonstrated, including separation of the two isomers PIC and nicotinic acid (NA) and the finding of a reduced PIC/QUIN ratio replicated in suicide attempters.

Metabolites of the kynurenine pathway of tryptophan (TRP) degradation (see Figure 1) have gained attention as putative pathophysiological mediators in various diseases [1–4]. In mammals, approximately 90% of dietary TRP is broken down into the tightly regulated enzymatic kynurenine pathway. Among the generated metabolites, several are neuroactive and have highly specialized effects that are tissue and cell specific [4].

**Figure 1.**
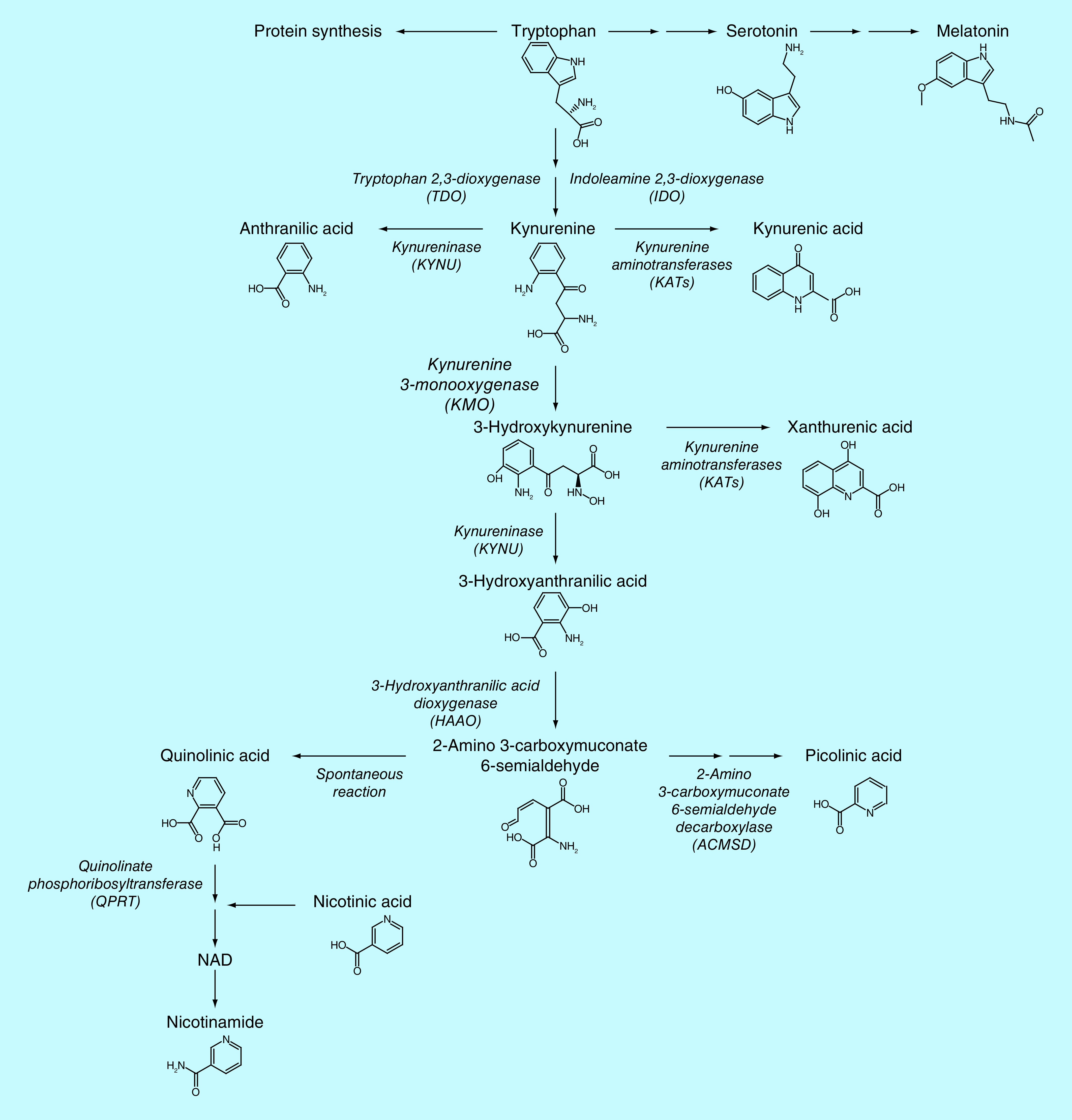
Kynurenine pathway of tryptophan degradation.

So far, kynurenic acid (KYNA) has been the most studied metabolite and its role in psychotic disorders and cognitive dysfunction is well-documented [1,2,5–8]. At low nanomolar concentrations, KYNA blocks the glycine site of the *N*-methyl-D-aspartate (NMDA) receptor [9,10], as well as the cholinergic α7 nicotinic receptor [11–14]. At higher micromolar concentrations, it blocks all three ionotropic glutamate receptors: the glutamate recognition site of the NMDA receptor as well as the kainate receptors and α-amino-3-hydroxy-5-methyl-4-isoxazol-propanoic acid (AMPA) receptors [9]. More recently, two targets engaging the immune system have been identified for KYNA: the former orphan G protein-coupled receptor-35 [15] and the aryl hydrocarbon receptor [16]. Besides targeting specific receptors, KYNA has the ability to scavenge hydroxyl, superoxide anions and other free radicals and therefore exerts antioxidant properties [17]. Quinolinic acid (QUIN), formed in the other branch of the kynurenine pathway, is an excitotoxic agonist at the NMDA receptor and can cause excitotoxicity and neuronal death at high concentrations [18–20]. Elevated concentrations of this metabolite have been associated with suicide attempts [21–23]. Picolinic acid (PIC) is suggested to possess a wide range of immunological, antiproliferative and neuroprotective properties [24]. Decreased levels of PIC, or an imbalance between the production of PIC and QUIN, has been suggested to be part of the neurobiological mechanisms underlying suicidal behavior [23]. This could indicate a reduced activity of the enzyme ACMSD, which generates PIC at the expense of QUIN, in individuals susceptible to develop suicidal behavior [23]. Such an enzymatic deficiency would make individuals vulnerable to produce more QUIN during situations of stress/inflammation, and could theroretically contribute to some individuals reacting with repeated suicidal behavior during life stressors, whereas other would not. Thus, low PIC could be used as a biomarker for vulnerability to suicidal behavior over time, whereas high CSF QUIN or a low PIC/QUIN ratio might indicate acute risk of suicide.

Several other metabolites have important biological functions. Thus, as KYNA, kynurenine is suggested to be a ligand of the human aryl hydrocarbon receptor [25] and xanthurenic acid (XA) as well as cinnabarinic acid are found to activate metabotropic glutamate receptors [26,27]. Furthermore, 3-hydroxykynurenine (3-HK) and 3-hydroxyanthranilic acid (3-HANA) are able to auto-oxidize and hence produce highly reactive hydroxyl radicals as well as H_2_O_2_ [28].

Besides having a role in psychiatric disorders, kynurenine metabolites have been implicated in various other diseases, such as cancer, cardiovascular, neurodegenerative, immunological and infectious disorders [4]. A vast majority of the studies published this far has focused on one or a few of the metabolites, since a robust method for quantitative and simultaneous detection of most of the metabolites with sufficient sensitivity and specificity has been lacking. There are several challenges when developing a single method for the detection of kynurenine metabolites, one of which is a vast span in physiological concentrations. Hence, CSF TRP is present in micromolar concentrations, whereas all other kynurenines are present in nanomolar concentrations [23,29–32]. High sensitivity is thus required for most kynurenines, while TRP which is present in high concentrations that may extend well beyond the linear dynamic range used for measurements of kynurenines. Another challenge is the closely related structures and physical properties of the pathway metabolites, including isomers, which challenges antibody-based methods of detection to distinguish structurally similar metabolites. As an example, it has been impossible to distinguish the isomers PIC and NA using antibody-based methods. TRP and kynurenine have been analyzed with HPLC with electrochemical detection, ultra high performance LC (UPLC), GC–MS and LC–MS [30,33–35]. KYNA has mostly been analyzed by HPLC with fluorescence detection [36–38], while QUIN and PIC have been analyzed by GC–MS [21,23,39]. Current interest in understanding the physiological and pathophysiological roles of kynurenines has highlighted the need to measure several metabolites simultaneously, hereby enabling a comprehensive understanding of the activity and regulation of the pathway in different physiological and disease states. The aims of the present study were to develop a highly sensitive biochemical analysis of TRP and multiple metabolites in the cerebrospinal fluid (CSF), to investigate their stability and to confirm our previous findings of aberrant CSF levels of QUIN and PIC in a small well-characterized validation cohort of suicide attempters relative to healthy controls using the novel method. Future studies can then use the method we have described here in order to generate robust, reproducible and comparable results, which will help refine the biomarkers for eventual clinical use.

## Material & methods

### UPLC–MS/MS method validation

#### Materials

##### Standards & Internal standards purchased

TRP, L-kynurenine (L-KYN), pyridine-2,3-dicarboxylic acid (QUIN), KYNA, PIC, nicotinamide (NAA), nicotinic acid (Niacin, NA), XA, 3-HK and 3-HANA – were all purchased from Sigma-Aldrich (MO, USA). The Internal standards (IS): TRP-*d*_3_. LYN-*d*_4_. QUIN-*d*_3_. [^13^C_6_]NAA, [^13^C_6_]NA, XA-*d*_4_. 3-HK-*d*_3_ and 3HA-*d*_3_ were obtained from Toronto Research Chemicals, Canada. KYNA-*d*_5_ and PIC-*d*_4_ were obtained from C/D/N Isotopes Inc. (Quebec, Canada). Solutions for the mobile phases: water, methanol and formic acid 99% were all LC–MS grade (Chromasolve, Honeywell, VWR International AB, Stockholm, Sweden).

### Standard solutions

Stock solutions of all unlabeled standards (L-KYN, QUIN, KYNA, PIC, NAA, NA, XA, 3-HK and 3-HANA) were prepared at 100 μM and TRP in 1000 μM in water and stored at -20°C. Calibrators were generated mixing all compounds in a final solution of 10 μM (TRP 100 μM). The calibrator mix was then aliquoted in volumes of 200 μl and stored in -80°C. Standard mix was thawed and serial diluted in water before each experiment. The IS stock solutions of all compounds were prepared at 5 μM with an exception of TRP-*d*_3_. which had the final concentration of 50 μM in water and stored in -80°C in aliquots of 350 μl. The IS working solution was prepared by dilution of IS stock (1:5) with 5% formic acid in water and stored at 4°C until analysis.

### Analysis with UPLC–MS/MS

The UPLC–MS/MS system used was a Xevo TQ-XS triple-quadrupole mass spectrometer (Waters, Manchester, UK) equipped with a Z-spray electrospray interface and a Waters Acquity UPLC I-Class FTN system (Waters, MA, USA). The MS was operated in electrospray-positive multiple reaction monitoring (MRM) mode. The system was operated using a source temperature of 150°C, capillary voltage of +3.0 kV, desolvation temperature 650°C, desolvation gas flow rate 1000 l/h and detector gain 1 was used. The *m/z* for the MRM transitions of each individual analyte, along with optimal cone voltages and collision energies were determined by manual tuning using the instrument’s built-in fluidics system (Masslynx 4.1 software). A 10 l/min flow of 100 ng/ml tuning solution was introduced to the mass spectrometer in combination with an LC flow of 0.2 ml/min and a composition of 20/80 mobile phase A/mobile phase B. The MRM transition providing the highest sensitivity was chosen as quantification trace for all compounds, except for TRP where the second most intense transition was chosen. This provided better linearity of the response over the calibration range, since the concentration of TRP in biological samples are significantly higher that for the other analytes in this method. The dwell times for the MRM channels were automatically calculated by the software, giving a desired number of 15–20 data points across the chromatographic peak. The precursor/product transitions for all compounds and belonging internal standards are shown in Table 1. The HPLC condition: the column was Acquity HSS T3 2.1*150 mm, 1.8 μm (Waters, Product Number [PN]: 186003540) and set to a temperature of 50°C. The two mobile phases were composed of A: 0.6% formic acid in water and B: 0.6% formic acid in methanol (UPLC grade). An isolator column (Waters, 2.1 × 50 mm column, PN: 186004476) was installed to retain contaminants from the mobile phase. The flow rate was set at 0.3 ml/min and the run time for each sample was 13.0 min. The autosampler was set at 4°C. Data processing was performed using Masslynx 4.1 software.

**Table 1.** Transitions and mass spectrometry parameters for all compounds and belonging internal standards.

Compound	Precursor ion mass	Product ion mass	Cone voltage (V)	Collision energy (eV)
NAA	123	78	25	16
		80^†^	25	16
PIC	123.9	78^†^	30	16
		96	30	16
NA	123.9	80^†^	25	16
		96	25	16
3-HA	154	108^†^	20	18
		136	20	10
QUIN	168.1	78	20	18
		124^†^	0	10
KYNA	190.1	116	30	29
		144^‡^	30	17
TRP	205.1	118	20	24
		146^†^	20	16
L-KYN	209.1	94^†^	20	12
		146	20	18
XA	206.1	160^†^	25	16
		188	25	10
3-HK	225.2	110.1^†^	14	16
		162.1	14	16
Internal standards	Precursor ion mass	Product ion mass	Cone voltage (V)	Collision energy (V)
NAA-^13^C_6_	129.1	101	20	16
PIC-*d_4_*	128	82	4	17
NA-^13^C_6_	130.1	57.1	32	16
3-HA-*d_3_*	157.0	83	24	24
QUIN-*d_3_*	171	81	20	18
KYNA-*d_5_*	195	121	28	26
TRP-*d_3_*	208.1	118.8	40	26
Kynurenine *d_4_*	213.2	94	30	15
XA-*d_4_*	210.1	192	25	10
3-HK-*d_3_*	228.2	163	14	16

† Quantifying ion.

‡ MS transition with the lowest response selected as quantifier ion.

3-HA: 3-Hydroxyanthranilic acid; 3-HK: 3-Hydroxykynurenine; KYNA: Kynurenic acid; L-KYN: L-Kynurenine; NA: Nicotinic acid; NAA: Nicotine amide; PIC: Picolinic acid; QUIN: Quinolinic acid; TRP: Tryptophan; XA: Xanthurenic acid.

### Sample preparation

Thirty microliter of human CSF sample, calibrator sample or Quality Control sample was mixed with 30 μl of IS working solution during 15 s in LC–MS Certified Clear Glass 12 × 32 mm vials (Waters, PN: 186005662CV) before transfer to an autosampler (set to 4°C) that injected 3 μl into the UPLC–MS/MS system, all samples included in the study were analyzed in duplicates.

### Method validation

The method was validated for selectivity, linearity, accuracy, precision and matrix effects in accordance with the guidelines of bioanalytical method validation from EMA and the US FDA. For chromatographic assays, recommended accuracy and precision variations are ±15% (LLOQ: ±20%) of nominal concentrations. Since we are analyzing endogenous components present in biological matrices, it is impossible to obtain totally analyte-free CSF for method validation. However, charcoal-treated matrices or other artificial matrices could be used as these do not contain most of the endogenous interferences that are present in the real matrices. Thus, in the present study, selectivity for all ten metabolites was investigated by comparing chromatograms of extracted blank CSF obtained from six different human samples spiked with a mix of all ten metabolites and IS to ensure that it was free of interference at the retention time of the compounds. The linearity was tested with a calibrator mix diluted in water in concentrations ranging between 0.1 and 250 nM (for TRP concentration was 0.1–25 μM). Each calibrator concentration and human CSF sample was analyzed in duplicates. The standard curve was calculated by 1/X-weighted least squares linear regression of standard curve calibrator concentrations and the peak area ratios of analyte to IS. An S/N ratio of three and S/N ratio of ten was used for estimating LOD and LOQ, respectively. Spiked CSF in two different concentrations (QA low and high: low: TRP 1.5 μM, L-KYN 50 nM, KYNA 2 nM, QUIN 20 nM, PIC 20 nM, NAA 10 nM, NA 2 nM, 3-HANA 2 nM, XA 2 nM, 3-HK 5 nM; high: TRP 15 μM, L-KYN 500 nM, KYNA 20 nM, QUIN 200 nM, PIC 200 nM, NAA 100 nM, NA 20 nM, 3-HANA 20 nM, XA 20 nM, 3-HK 50 nM) was used in order to test the accuracy and precision of the assay. Accuracy is presented as percentage recovery (100 × [measured C_spiked_-C_nonspiked_/C_spiked_]) and the assay precision is presented as percent relative standard deviation (STDEV [Data Range]/AVERAGE [Data Range] *100) and was calculated from repeated measurements within the same experiment (intra-assay, n = 6, during 20 h with samples stored in sample manager at 4°C) or from three different experiments running over 2 days (interassay). The matrix effect was calculated from the area of the IS in CSF (n = 6) in relation to the response in a prepared sample of pure water (n = 6), as (Area [CSF]/Area [water] - 1) *100% (see Table 1).

### Stability tests

The bench-top stability was evaluated using six separate human CSF samples stored at room temperature for up to 4 h after first thawing (covering normal laboratory handling of sample analysis). The freeze–thawing stability was evaluated using six human CSF samples frozen at -80°C, then thawed to room temperature for four cycles with 24 h between. In order to investigate stability before and after the first freezing cycle, we used CSF samples from four different subjects. These CSF samples were analyzed directly after lumbar puncture, before freezing and next after 24 h at room temperature before any freezing, then again after one freezing cycle (covering normal handling for clinically collected CSF). Percent stability for each analyte is given as the mean percent stability of the six individual CSF ± standard deviation (SD) at the given hour.

### Stability cohort

In December 2017 and in March 2019, ten multiple sclerosis (MS) patients at the MS outpatient clinic at the Karolinska University Hospital were asked to participate in the study: patients should meet the following criteria: being 18–55 years of age. No known major somatic or psychiatric diagnoses besides MS. No psychiatric or psychotropic medication, including glucocorticoids, within 90 days of sampling. CSF from the six patients enrolled in December 2017 was analyzed following storage in -80°C until analysis (February 2018) and used for testing the stability following repeated freezing and thawing cycles as well as following storage at room temperature for up to 4 h. CSF from the four patients enrolled in 2019 was used for investigating the impact of the first freezing and thawing cycle, in other words, that the CSF was analyzed within 30 min after the lumbar puncture and before putting the samples into the freezer. The next day, following 24 h in either -80°C or at room temperature, the same samples were analyzed again.

## Biological validation

### Inclusion criterium: definition of suicide attempt

The inclusion criteria for the patient group was that they had performed a suicide attempt with a clear intent to die. A suicide attempt was defined as “situations in which a person has performed an actually or seemingly life-threatening behavior with the of jeopardizing his/her life” [40]. The patients needed to explicitly state the intent in the interviews with the study physician in order to be enrolled. Patients could belong to any diagnostic category, aligned with the concept that suicidality is a cross-diagnostic phenomenon (CITE the Diagnostic and Statistical Manual of Mental Disorders [DSM] V) with a common biological denominator.

### Cohort description

Thirteen patients (seven men, six women; mean age 39.2 ± 15.6 [SD] years; median BMI 26, range 19–51; BMI data missing for one patient) were enrolled following admission to Lund University Hospital after a suicide attempt (see above for definition). Thirteen healthy controls (five men, eight women, mean age 40.4 ± 14.6 [SD] years; median BMI 24, range 19–30) were recruited via the population registry; and were prescreened via a phone interview before being invited to enroll in the study and subsequent diagnostic interviews (Table 5). The study enrolled patients and controls between 2009 and 2012. Controls did not suffer from any previous or ongoing psychiatric condition or substance abuse and were somatically healthy. Eleven of the 13 patients were on medical treatment when enrolled in the study, the most common medications were painkillers (n = 6), antipsychotics (n = 5), antidepressants (n = 8), sleep aids (n = 6), mood stabilizers (n = 3), anxiolytics (n = 4) (for list of all drugs used see Supplementary Table 1). Subjects were allowed to take pain killers (n = 2 controls; 6 suicide attempters), oral contraceptives (n = 1; controls) and nonprescription allergy medications (n = 2; controls). Lumbar puncture was carried out on all healthy controls and patients as described below. Briefly after the suicide attempt, a psychiatrist diagnosed all patients according to the DSM IV. The diagnoses were set by the psychiatrist within days after the suicide attempt and after an approximately 2-h-long structured interview using the Comprehensive Psychiatric Rating Scale [41] and the Structured Clinical Interview for DSM Disorders (SCID I and II).

### Somatic examination

All patients and controls in both cohorts underwent a general physical examination and a complete medical history was taken. We measured the subjects temperature and screened the blood for erythrocyte sedimentation rate, C-reactive protein and white blood cell count to identify any participants with infections. All measurements were within the normal reference levels for these parameters.

### Collection & storage of CSF from all cohorts

We used a standardized protocol to draw CSF from the L4–L5 interspace, between 8 AM and 11 AM after a night of bed rest and fasting. We kept all samples on ice and immediately aliquoted and froze them at -80°C, except for the samples that were used for stability testing. The suicidal patients were psychiatric inpatients, whereas the controls and MS patients had spent the night at home. CSF from MS patients was analyzed within 2 months from collection, whereas CSF from the suicidal patients and the healthy controls was analyzed within 6–9 years after collection.

### Statistics

The statistical analysis were performed using IBM SPSS Statistics 20.0 (IBM SPSS, IL, USA) and graphs were performed with GraphPad Prism 8 and SPSS 25 for Mac. The nonparametrical two-tailed Mann–Whitney U-test was used to compare age, BMI and concentrations of TRP, L-KYN, KYNA, 3-HK and NAA, between healthy controls and suicide attempters. Since we have previously published, and our hypothesis is, that QUIN and the L-KYN/TRP ratio are increased and that PIC and the PIC/QUIN ratio are reduced in suicide attempters, we used one-tailed Mann–Whitney U-test when comparing the CSF levels of these metabolites as well as the ratios between healthy controls and suicide attempters. Data are expressed as mean ± SD. Spearman rank correlation analysis was used to analyze correlations between markers. Statistical significance was considered when p < 0.05.

## Results

### Method validation

#### Linearity, sensitivity & selectivity of the method

**Figure 2.**
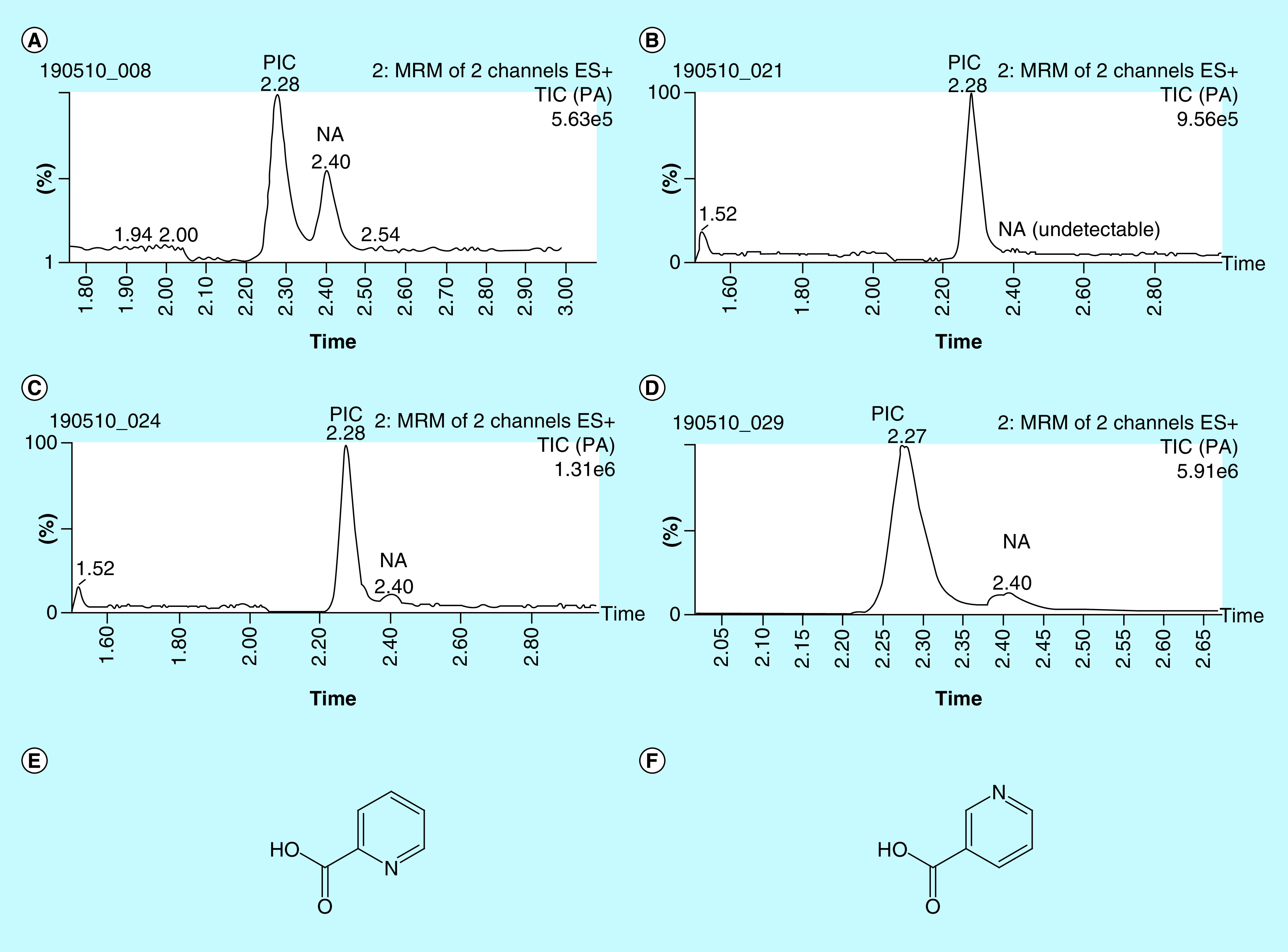
Chromatograms of the two isomers picolinic acid and nicotinic acid. Representative chromatograms showing separation of the two isomers PIC and NA in **(A)** standard of PIC and NA 1 nM, **(B)** unspiked cerebrospinal fluid (CSF), **(C)** CSF spiked with 20 nM PIC and 2 nM NA, **(D)** CSF spiked with 200 nM PIC and 20 nM NA, chemical structure of PIC **(E)** and chemical structure of NA **(F)**. ES+: Positive electrospray ionisation mass spectrometry; MRM: Multiple reaction monitoring; NA: Nicotinic acid; PIC: Picolinic acid; TIC: Total ion current.

The calibration curve of all TRP and nine metabolites measured using stable isotope-labeled IS indicated *good linearity* within the concentration range tested (0.1–250 nM; TRP 0.1–250 μM) (Table 2). The correlation coefficient R2 of the regression equations ranged between the value of 0.98–0.99 for all analytes with an exception of 3-HANA that had an R2 of 0.96. The S/N ratios of LLOQ samples were in the range for covering stable detection (>10) and thus entail a good sensitivity of the assay for detection of TRP, L-KYN, QUIN, KYNA, PIC, NAA and 3-HK. Three of the metabolites, NA, XA and 3-HANA, were detected in fewer than 15% of all CSF samples measured (n = 35) and therefore were excluded from further analyses. The specificity of the method for separation of the isomers PIC and NA is presented in Figure 2. By using the current method, we have a clear separation of the two peaks, PIC and NA.

**Table 2.** Intra-assay (six repeated analyses within one experiment during 20 h with samples stored in sample manager) and interassay validation.

Compound	Linearity (R^2^)	LOD–LLOQ	% Matrix effects	Intra-assay (n = 6 during 20 h in +4°C)				Interassay (n = 3)			
				Accuracy (% of target)		Precision (RSD%)		Accuracy (% of target)		Precision (RSD%)	
				Conc A	Conc B	Conc A	Conc B	Conc A	Conc B	Conc A	Conc B
TRP	0.996	10.0–10.0	-8.4	102	108	1	1	101	107	2	2
L-KYN	0.997	0.1–0.25	-4.1	96	115	1	1	95	114	1	3
KYNA	0.998	0.1–0.5	-12.1	102	106	1	1	102	106	2	1
QUIN	0.998	2.5–5.0	3.3	95	97	2	1	94	97	1	4
PIC	0.979	1.0–5.0	12.5	112	89	3	5	113	87	4	1
NAA	0.996	2.5–10	2.4	94	93	3	1	92	92	14	3
NA	0.997	1.0–5.0	-4.0	101	96	9	1	97	95	2	1
3-HANA	0.963	1.0–5.0	-3.9	136	97	4	2	140	97	8	8
XA	0.989	2.5–10.0	6.0	95	85	2	2	97	86	7	3
3-HK	0.997	0.25–1.0	23.4	94	91	1	2	95	92	1	2

Intra-assay (six repeated analyses within one experiment during 20 h with samples stored in sample manager) and interassay (three independent experiments within 2 days, all) validation results. Concentration A: two-times endogenous levels, B: ten-times endogenous CSF levels for all metabolites in blank CSF. Matrix effects calculated from ratio of IS area in matrix/IS are in blank sample). LOD–LLOQ concentration of TRP is in μM, all other metabolite (L-KYN, KYNA, QUIN, PIC, NAA, NA, 3-HANA, XA and 3-HK) concentrations are in nM.

3-HANA: 3-Hydroxyanthranilic acid; 3-HK: 3-Hydroxykynurenine; CSF: Cerebrospinal fluid; IS: Internal standard; KYNA: Kynurenic acid; L-KYN: L-Kynurenine; LLOQ: Lower limit of quantification (S/N ratio of ten); LOD: Limit of detection (S/N ratio of three); NA: Nicotinic acid; NAA: Nicotine amide; PIC: Picolinic acid; QUIN: Quinolinic acid; RSD: Relative standard deviation; TRP: Tryptophan; XA: Xanthurenic acid.

#### Inter- & intra-assay precision & accuracy

Accuracy and precision were evaluated at two different concentrations of spiked CSF, covering the range of concentrations found in CSF. Intra-assay accuracy was stable for the majority of metabolites (9/10) with small variations 85 and 112% of the target (Table 2). Intra-assay precision ranged between 1 and 9% relative standard deviation (RSD). The interassay accuracy varied between 86 and 113%, while the interassay precision ranged between 1 and 14% RSD (Table 2). 3-HANA was the only metabolite that had unsatisfactory variation in both intra- and interassay accuracy (134 and 140%, respectively, of target).

#### Matrix effects

As endogenous concentrations of analytes are present in matrices, evaluated CSF matrix effects were determined with IS only and the matrix factor of IS calculated, in accordance with EMA and US FDA and guidelines. Matrix effects were found to be less than 13% in all metabolites tested with an exception for 3-HK that had 23% (Table 2).

#### Stability

The stability of kynurenine metabolites were tested in CSF from six subjects with MS. Mean baseline concentrations in these six subjects were TRP (1.5 ± 0.5 μM), kynurenine (60.2 ± 30.6 nM), KYNA (2.4 ± 0.8 nM), QUIN (23.6 ± 19.7 nM), PIC (14.5 ± 3.0 nM), 3-HK (3.1 ± 1.2 nM) and NAA (22.8 ± 20.9 nM). The percent stability of TRP, kynurenine, KYNA, QUIN, PIC and NAA ranged between 89 and 113% in samples stored at room temperature for 30 min, 1, 2, 3 and 4 h (Table 3). The average decrease after repeated freeze–thaw cycles was below 15% (Table 4). Storage at room temperature for up to 4 h did not affect the levels of any metabolite.

**Table 3.** Percent stability (mean ± SD) of kynurenine analytes at room temperature after 30 min, 1, 2, 3, 4 and 24 h.

Compound	Baseline mean ± SD	% change					
		30 min	1 h	2 h	3	4 h	24 h
TRP	1.5 ± 0.5 μM	105 ± 4.9	105 ± 4.9	99 ± 4.9	101 ± 4.9	105 ± 4.9	95 ± 2.4
L-KYN	60.2 ± 30.6 nM	95 ± 4.1	96 ± 4.1	93 ± 4.1	96 ± 4.0	100 ± 2.7	95 ± 8.8
KYNA	2.4 ± 0.8 nM	113 ± 4.9	102 ± 4.9	89 ± 4.9	100 ± 4.7	109 ± 4.7	99 ± 1.6
QUIN	23.6 ± 19.7 nM	103 ± 5.9	106.2 ± 1.6	100 ± 4.6	101 ± 4.4	108 ± 2.5	111 ± 7.3
PIC	14.5 ± 3.0 nM	98 ± 4.8	101 ± 4.2	96 ± 4.5	99 ± 4.0	104 ± 4.6	98 ± 4.9
PIC/QUIN ratio	0.8	104.4 ± 9.9	95.4 ± 5.1	96.4 ± 4.3	98.1 ± 5.6	96.6 ± 5.1	92,7 ± 4.3
NAA	22.8 ± 20.9 nM	100 ± 6.1	102 ± 1.9	96 ± 6.4	103 ± 7.2	111 ± 7.2	104 ± 5.9
3-HK	3.1 ± 1.2 nM	99 ± 5.1	102 ± 2.9	97 ± 6.0	97 ± 4.1	102 ± 2.7	75 ± 4.0

Compared with 100% at t = 0, mean ± SD from 4 to 6 individual CSF samples. For baseline and all freeze–thaw cycles, n = 6.

3-HK: 3-Hydroxykynurenine; CSF: Cerebrospinal fluid; KYNA: Kynurenic acid; L-KYN: L-Kynurenine; NAA: Nicotine amide; PIC: Picolinic acid; QUIN: Quinolinic acid; SD: Standard deviation; TRP: Tryptophan.

**Table 4.** Percent stability (mean ± SD) of tryptophan metabolites after 1–5 freeze–thaw cycles.

Compound	Baseline mean ± SD	% change			
		Thawing 2	Thawing 3	Thawing 4	Thawing 5
TRP	1.5 ± 0.5 μM	104 ± 4.9	100 ± 4.9	99 ± 4.9	101 ± 4.9
L-KYN	60.2 ± 30.6 nM	98 ± 4.1	94 ± 4.1	94 ± 4.07	95 ± 4.1
KYNA	2.4 ± 0.8 nM	100 ± 4.9	96 ± 4.9	107 ± 4.9	101 ± 4.9
QUIN	23.6 ± 19.7 nM	105 ± 1.5	101 ± 1.5	102 ± 5.9	100 ± 5.9
PIC	14.5 ± 3.0 nM	101 ± 4.7	97 ± 4.7	99 ± 4.6	101 ± 6.3
PIC/QUIN ratio	0.8	101.8 ± 7.1	105.7 ± 10.7	105.9 ± 9.2	108.1 ± 8.4
NAA	22.8 ± 20.9 nM	101 ± 2.3	98 ± 2.0	98 ± 7.6	105 ± 6.6
3-HK	3.1 ± 1.2 nM	103 ± 2.9	97 ± 1.7	97 ± 1.7	96 ± 1.7

Compared with 100% at t = 0, mean ± SD from 4 to 6 individual CSF samples. For baseline and all freeze–thaw cycles, n = 6.

3-HK: 3-Hydroxykynurenine; CSF: Cerebrospinal fluid; KYNA: Kynurenic acid; L-KYN: L-Kynurenine; NAA: Nicotine amide; PIC: Picolinic acid; QUIN: Quinolinic acid; SD: Standard deviation; TRP: Tryptophan.

The stability of kynurenine metabolites following the very first freeze–thaw cycle was analyzed in four additional subjects with MS. Mean baseline concentrations in fresh CSF, analyzed within 30 min after lumbar puncture and before storing the samples in the -80°C freezer, were TRP (1.3 ± 0.1 μM), kynurenine (67.7 ± 57.7 nM), KYNA (2.0 ± 0.4 nM), QUIN (22.0 ± 3.6 nM), PIC (9.2 ± 4.2 nM), 3-HK (4.4 ± 3.2 nM) and NAA (26.2 ± 10.4 nM).

The percent stability after one freeze–thaw cycle ranged between 93 and 104% TRP (102 ± 3 %), kynurenine (104 ± 4 %), KYNA (98 ± 2 %), QUIN (99 ± 2 %), PIC (100 ± 3 %), 3-HK (93 ± 4%) and NAA (93 ± 8 %). Storage at room temperature for 24 h decreased 3-HK levels with 25% (3.7 ± 2.5), but did not affect the levels of any other metabolite analyzed.

### TRP & its metabolites in the CSF of healthy controls & suicide attempters

#### Demographics

No differences were found regarding biological sex (13 healthy controls: five male and eight female, 13 suicide attempters: seven male and six female), age (healthy controls: 40.4 ± 14.6, suicide attempters: 39.2 ± 15.6) and BMI (healthy controls: median BMI 24, range 19–30, suicide attempters: BMI 26, range 19–51) between healthy controls and suicide attempters. Mean total SUAS score among suicide attempters were 43.8 ± 17.0.

#### TRP & six of its metabolites are detectable in the CSF

**Figure 3.**
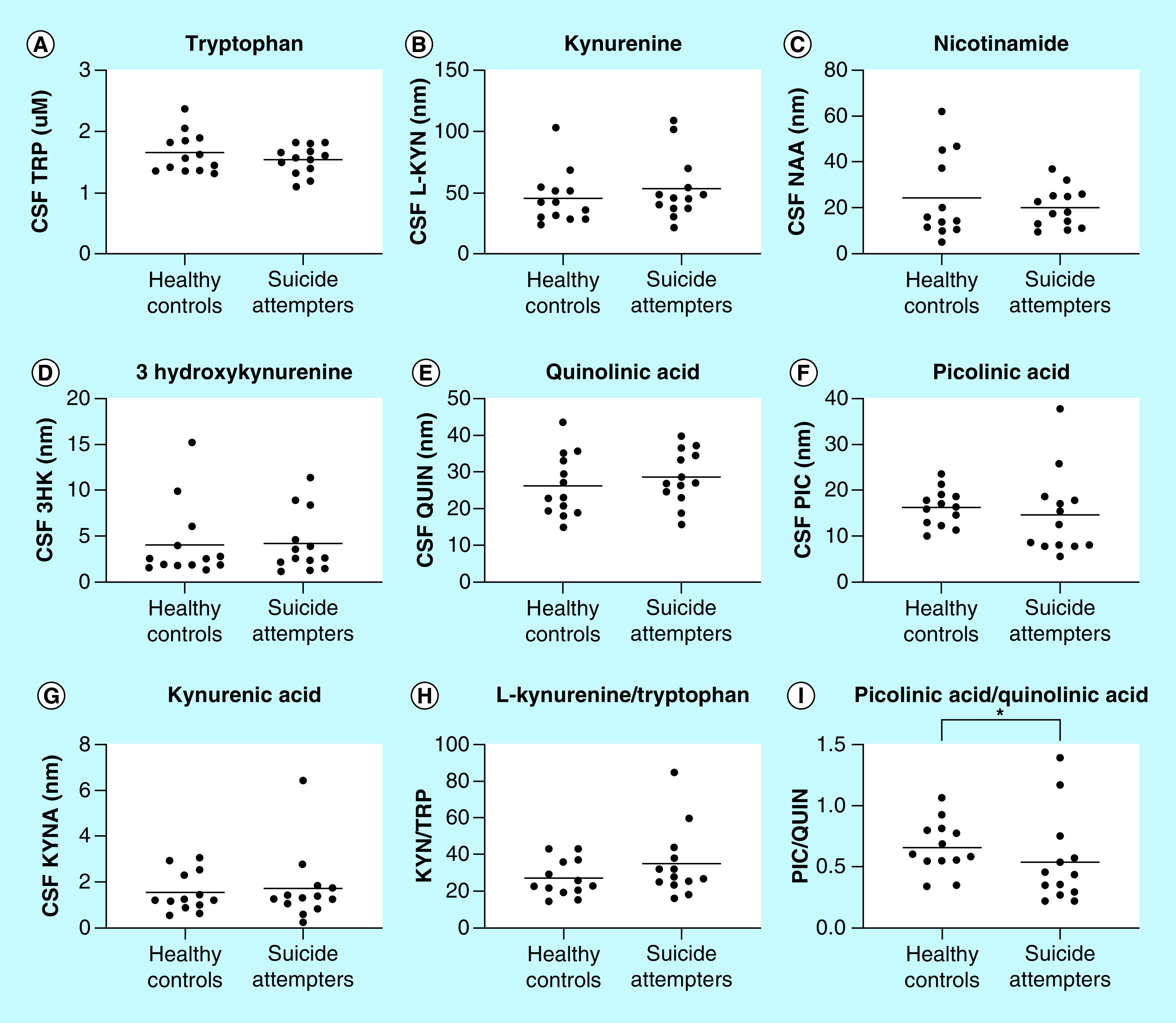
Cerebrospinal fluid levels of kynurenines in suicide attempters and healthy volunteers. Levels of **(A)** tryptophan, **(B)** kynurenine, **(C)** nicotinamide, **(D)** 3-hydroxykynurenine, (**E)** quinolinic acid, **(F)** picolinic acid, **(G)** kynurenic acid, **(H)** ratio of kynurenine/tryptophan and (**I)** ratio of picolinic acid/quinolinic acid in the CSF of suicide attempters and healthy controls. Each point represents the concentration of a single CSF sample in units of nM and mean is displayed in all graphs but for tryptophan concentration where the unit is in μM. Statistical differences between healthy controls and suicide attempters are determined using two-tailed Mann–Whitney test, *p < 0.05. CSF: Cerebrospinal fluid; KYNA: Kynurenic acid; PIC: Picolinic acid; QUIN: Quinolinic acid.

TRP and six of its metabolites were successfully detectable in all CSF samples from healthy controls and suicide attempters, see Figure 3 (TRP: healthy controls 1.7 ± 0.3 μM and suicide attempters 1.5 ± 0.2 μM, L-KYN: healthy controls 45.1 ± 21.5 nM and suicide attempters 52.6 ± 25.9 nM, KYNA: healthy controls 1.6 ± 0.9 nM and suicide attempters 1.7 ± 1.5 nM, 3-HK: healthy controls 4.1 ± 4.1 nM and suicide attempters 4.2 ± 3.3 nM, NAA: healthy controls 24.4 ± 18.5 nM and suicide attempters 20.2 ± 8.7 nM, PIC: healthy controls: 16.3 ± 3.9 nM and suicide attempters: 14.7 ± 9.1 nM, QUIN: healthy controls: 26.4 ± 8.5 nM and suicide attempters: 28.7 ± 7.3 nM).

#### Decreased PIC/QUIN ratio in suicide attempters

The CSF PIC/QUIN ratio was found to be decreased in the suicide attempters compared with healthy controls (healthy controls: 0.7 ± 0.2 and suicide attempters: 0.5 ± 0.4: p = 0.03). Furthermore, the differences in the CSF levels of PIC and QUIN (see above) as well as the L-KYN/TRP ratio between healthy controls and suicide attempters were all in the hypothesized directions, although without reaching statistical significances (PIC: p = 0.11; QUIN: p = 0.19; L-KYN/TRP: healthy controls: 27.1 ± 9.9 and suicide attempters: 34.9 ± 18.9; p = 0.11).

#### Correlations between L-KYN & KYNA & between L-KYN & QUIN

We detected a significant positive correlation of L-KYN to both KYNA (r = 0.7 p = <0.0001) and QUIN (r = 0.5447, p = 0.001), but not to 3-HK (r = -0.06, p = 0.7) in CSF from all subjects (n = 32, controls, suicidal patients and MS patients). 3-HK did not show any correlation with QUIN (r = -0.01, p = 0.9) in this CSF material.

**Table 5.** Study population demographics

Demographics	Controls (n = 13)	Suicidal attempters (n = 13)	p-value^2^
Mean age in years (±SD)	40.4 (±14.6)	39.2 (±15.6)	0.49
Percentage male sex	41.7%	58.3%	
Median BMI (IQR)	24 (19–30)	26 (19–51)	0.0225

IQR: Interquartile range; SD: Standard deviation.

## Discussion

Simultaneous detection of multiple kynurenine pathway metabolites is important to further understanding of their role(s) in different disease states. In the present study, we have developed a new protocol with high accuracy and precision for the quantification of multiple CSF TRP metabolites using UPLC–MS/MS. In addition, this method also clearly separated the two isomers, PIC and NA. Seven metabolites: TRP, L-KYN, NAA, 3-HK, QUIN, PIC, KYNA, were reliably detectable and measured in the CSF. Thus, this is the first UPLC–MS/MS study showing detectable CSF levels of PIC, at the same time visualizing the isomer NA as a clearly separate peak (see Figure 2). To our knowledge, one study analyzing both PIC and NA with UPLC–MS/MS has previously been published. However, that method was not sensitive enough for CSF measurements [42]. PIC, NA and QUIN can also be detected with GC/MS [39]; however, this method uses esterification of the acids in order to enable separation and detection, the measured products are thus conjugated acids and not the acids themselves. This might explain why previous studies, using GC/MS, seem to find somewhat higher concentration of PIC than we do in the present manuscript.

Several kynurenine metabolites, such as PIC, QUIN and KYNA, are suggested as putative biomarkers that may be used in the diagnostics for several diseases. As such, in order to develop robust biomarkers for clinical application, it is of critical importance to have information about the stability of the metabolites over time and following freezing and thawing. Indeed, in some clinical situations, samples may be standing on the bench for some time during the sampling procedure or in the laboratory during the biochemical analysis. Furthermore, CSF used in clinical research might be sparse, and therefore it might be useful to know whether the levels of metabolites are stable over numerous cycles of freezing and thawing. For simulation of these conditions, we explored the effects of storage in room temperature (22°C) as well as the influence of several repeated freeze–thaw cycles on the concentrations of the kynurenine metabolites. The result of the stability test clearly demonstrates that all detectable metabolites in CSF are stable up to 4 h in room temperature and do not degrade following multiple freeze–thaw cycles. However, the concentrations of 3-HK was found to be decreased following long-term, up to 24 h, storage in room temperature.

In the present study, efforts were also made to investigate if the very first freeze–thaw cycle affected the concentration of the metabolites. Thus, CSF samples were analyzed within 30 min after the lumbar puncture and again following 24 h in the -80°C freezer. We found that the first freeze–thaw cycle did not affect the levels of any detected metabolite (TRP, L-KYN, NAA, 3-HK, QUIN, PIC, KYNA).

We have previously used GC–MS to analyze the levels of PIC and QUIN in blood and CSF from several cohorts of suicide attempters and controls [23]. Our results showed that CSF QUIN was elevated during acute suicidal episodes, possibly on the basis of a reduced activity of the enzyme ACMSD, which generates PIC at the expense of QUIN. In order to validate the robustness and reproducibility of the novel method in a clinical research context, we here analyzed kynurenines in the CSF from a novel small cohort of suicide attempters and healthy controls. As in the stability cohort, seven kynurenine metabolites were detectable in the CSF. We were able to replicate our original finding [23], of a reduced PIC/QUIN ratio in suicide attempters using the novel method, which speaks to its robustness and future clinical usefulness. In order to further develop kynurenine metabolites including QUIN and PIC as biomarkers for psychiatric conditions such as suicidality, a comprehensive analysis in several large patient cohorts need to be undertaken next.

In the present study, we also discovered a strong positive correlation between L-KYN and QUIN as well as between L-KYN and KYNA, indicating that *de novo* production of these metabolites is highly dependent on the availability of kynurenine in CSF. Surprisingly, 3-HK did not follow this pattern. Furthermore, we could not find any correlation between 3-HK and QUIN, even though both these two metabolites are produced by the same rate-limiting enzyme kynurenine monooxygenase. These data encourage further investigation of the pattern of metabolism in the kynurenine pathway not only during normal conditions but also in an inflammatory state.

Collectively, the method described here has several advantages over previous protocols for detecting multiple kynurenine metabolites and as such represents a valuable tool for advancing our knowledge on the role of kynurenine pathway in different disease states.

## Future perspective

Kynurenine metabolites are gaining attention as modulators and key players of several disorders, including cancer, cardiovascular, neurodegenerative, psychiatric, immunological and infectious disorders. Indeed, metabolites of the kynurenine pathway have the potential to serve as biomarkers in future clinical settings. It is, therefore, pivotal to develop a quantification method for simultaneously analysis of multiple metabolites with high precision. In addition, the stability of metabolites following several freezing and thawing cycles and/or different times in room temperature is of a importance for the future of biomarker research. In the present study, we have developed a sensitive assay for quantification of multiple kynurenine pathway metabolites. Importantly, the method clearly separate PIC from its isomer niacin. We were also able to confirm our previous report showing that the CSF ratio of two of the metabolites, PIC/QUIN, is decreased in suicide attempers. The purpose of including this small pilot of suicide attempters in the current manuscript was to validate the robustness of the biomarkers. Ongoing studies in our lab utilize large cohorts with several populations of patients and aim to find cutoff values to define the use of these biomarkers in the clinic. We believe that this method will be a corner stone for future analysis of kynurenine pathway metabolites and pave the way for the development of clinical biomarkers for psychiatric disorders, in particular suicidality.

**Summary points**

**Background**

- Kynurenine metabolites are modulators of and key players in several disorders.
- There is a need for a high sensitive and high precision method for simultaneous analysis of multiple kynurenine metabolites.

**Experimental**

- An UPLC–MS/MS method was developed in order to measure ten cerebrospinal fluid tryptophan metabolites with high sensitivity and able to separate the two isomers, picolinic acid (PIC) and NIC.
- The method was developed following the US FDA guidelines for bioanalytical analysis testing matrix effects, accuracy and precission.

**Results & discussion**

- The developed method had a small inter- and intra-assay variation.
- The linearity and the sensitivity were good, as well as the specificity of NIC and PIC chromatogram separation.
- All metabolites, except 3-hydroxyanthranilic acid were stable for several freeze–thaw cycles as well as for storing in room temperature up to 4 h.
- A reduced PIC/quinolinic acid ratio in suicide attempters was confirmed.
