# Supplementary material for: A novel, robust method for quantification of multiple kynurenine pathway metabolites in the cerebrospinal fluid
Source: Bioanalysis. 2020 Mar 25;12(6):379–92. doi: 10.4155/bio-2019-0303 (PMC9472175; doi:10.4155/bio-2019-0303)
Supplement: Supplementary file 1 [file bio-12-379-s1.docx]

| Number of subjects using | Medication |
| --- | --- |
| 4 | Paracetamol |
| 3 | Alimemazine |
| 3 | Propiomazine |
| 3 | Quetiapine |
| 2 | Naproxen |
| 2 | Ibuprofen |
| 2 | Sertraline |
| 2 | Omeprazole |
| 1 | Prednisolone |
| 1 | Phenylpropanolamine |
| 1 | Simeticone |
| 1 | Diclofenac |
| 1 | Levothyroxine |
| 1 | Disulfiram |
| 1 | Venlafaxine |
| 1 | Paroxetine |
| 1 | Reboxetine |
| 1 | Hydroxyzine |
| 1 | Zolpidem |
| 1 | Citalopram |
| 1 | Dipropylacetate |
| 1 | Clonazepam |
| 1 | Levopromazine |
| 1 | Zopiclone |
| 1 | Pregabalin |
| 1 | Mirtazapine |
| 1 | Nitrazepan |
| 1 | Lamotrigine |
| 1 | Agomelatine |
| 1 | Litihum |
| 1 | Metoprolol |
